# Supplementary material for: AffyMAPSDetector: a software tool to characterize Affymetrix GeneChip™ expression arrays with respect to SNPs
Source: BMC Bioinformatics. 2007 Jul 30;8:276. doi: 10.1186/1471-2105-8-276 (PMC1959249; doi:10.1186/1471-2105-8-276)
Supplement: Additional file 13 — AffyMAPSDetector v1 source code. AffyMAPSDetector source code. [file 1471-2105-8-276-S13.zip › AffyMAPSDetector_v1_src/doc/affychip/snpdetector/util/package-tree.html]

affychip.snpdetector.util Class Hierarchy


|  |  |  |  |
| --- | --- | --- | --- |
| **Overview** \| **Package** \| Class \| Use \| **Tree** \| **Deprecated** \| **Index** \| **Help** | | |  |
| **PREV**   **NEXT** | **FRAMES**    **NO FRAMES**     **All Classes** |  |  |


---


## Hierarchy For Package affychip.snpdetector.util

**Package Hierarchies:**: All Packages

---

## Class Hierarchy

- class java.lang.Object
  - class affychip.snpdetector.util.**ColorGradient**- class java.awt.Component (implements java.awt.image.ImageObserver, java.awt.MenuContainer, java.io.Serializable)
      - class java.awt.Container
        - class java.awt.Window (implements javax.accessibility.Accessible)
          - class java.awt.Dialog
            - class javax.swing.JDialog (implements javax.accessibility.Accessible, javax.swing.RootPaneContainer, javax.swing.WindowConstants)
              - class affychip.snpdetector.util.**GeneAnnotLoaderWizard** (implements jwf.WizardListener)
            - class java.awt.Frame (implements java.awt.MenuContainer)
              - class javax.swing.JFrame (implements javax.accessibility.Accessible, javax.swing.RootPaneContainer, javax.swing.WindowConstants)
                - class affychip.snpdetector.util.**ComplementDnaSeq**- class affychip.snpdetector.util.**DuplicateRemover**- class affychip.snpdetector.util.**PmMmHeatMapViewer**
      - class affychip.snpdetector.util.**GuiUtils**- class affychip.snpdetector.util.**PmMmHeatmapViewerModel**- class affychip.snpdetector.util.**PmMmHeatmapViewerModel.Row** (implements java.lang.Comparable)- class affychip.snpdetector.util.**PmMmHeatmapViewerModel.RowComparatorForDataSorting** (implements java.util.Comparator)- class affychip.snpdetector.util.**PmMmMatrixLoader**

---


|  |  |  |  |
| --- | --- | --- | --- |
| **Overview** \| **Package** \| Class \| Use \| **Tree** \| **Deprecated** \| **Index** \| **Help** | | |  |
| **PREV**   **NEXT** | **FRAMES**    **NO FRAMES**     **All Classes** |  |  |


---
